# Deprexis® Acceptability study in REal life (DARE): study design

**DOI:** 10.1192/j.eurpsy.2023.1820

**Published:** 2023-07-19

**Authors:** O. Amiot, A. H. Clair, P. Courtet, E. Fakra, V. Narboni, F. Gheysen, E. Haffen, D. Drapier, L. Lecardeur

**Affiliations:** 1 Groupe Hospitalier Paul Guiraud, Boulogne Billancourt; 2 ICM - Paris Sorbonne Université, Paris; 3 CHU, Montpellier; 4 CHU, Saint Etienne; 5 Ethypharm Digital Thérapy, Paris; 6 cabinet libéral, Caen; 7 CHU, Besancon; 8 CH Guillaume Regnier, Rennes; 9 consultant, Nice, France

## Abstract

**Introduction:**

Depression is a leading cause of disability, worldwide. Recently, WHO highlighted the negative impact of recent crises (COVID-19 pandemic, war in Ukraine, economic crisis).

Although most international guidelines recommend psychotherapies as first-line treatment of depression, access remains scarce in France due to limited availability of trained clinicians (notably those with CBT certification), high cost for patient in a context of non-reimbursement and fear of stigmatization (Coldefy M. HCAA, 2022/04,19). Therefore, online blended psychological treatment such as deprexis® could increase access to care for people with depression. It presents several advantages such as easy access, scalability, and a proven efficacy (Twomey et al. PLoS One. 2020;15(1):e022810).

**Objectives:**

This study aims to test real-life acceptability of deprexis® for people with depression in France outside a reimbursement pathway.

Primary objective of this cross-sectional study is to measure acceptability of deprexis® a new digital therapy in France.

Questionnaire includes acceptability of deprexis® assessed with patient willingness to complete deprexis® course, reasons of refusal, when needed, demographics and depression characteristics.

The secondary objectives are to study 1/ acceptability according to type of center (Hospital based, Community Based or private practice) and type of practitioners (psychiatrists or psychologists), 2/ differences in acceptability according to severity’s level (evaluated with PHQ 9), 3/ differences in acceptability according to administration or not of a treatment (including psychotherapy), 4/ differences in acceptability according to prescriber’s profile (age, sex, place and type of practice), 5/ identification of reasons for refusal , and 6/ analyze refusal rate over time.

**Methods:**

DARE is as a cross-sectional study in which deprexis® is suggested to any patient meeting the inclusion criteria over the fixed inclusion period June-December 2022

Inclusion criteria are: 1/ depression, 2/ age between 18 and 65 years, 3/ speak French sufficiently, 4/ access to Internet with a device to connect to deprexis® platform.

Exclusion criteria are diagnosis of bipolar disorder, psychotic symptoms and/or suicidal thoughts during the current episode.

All investigators received a video-based training on deprexis® before inclusion to make sure they all have same level of information and understanding on the program.

**Results:**

The study is currently recruiting. Data will be available for EPA congress.

**Conclusions:**

It is a first time a digital therapy is completing the current therapeutic options for the treatment of depression in France. Acceptability of this innovation by both patients and Healthcare providers is a first step.

DARE may allow to have a better understanding of the acceptability of a digital therapy in the treatment of depression in France and identify the different factors influencing it in a natural setting.

**Disclosure of Interest:**

O. Amiot Consultant of: Ethypharm Digital Therapy, A. H. Clair Consultant of: Ethypharm Digital Therapy, P. Courtet Consultant of: Ethypharm Digital Therapy, E. Fakra Consultant of: Ethypharm Digital Therapy, V. Narboni Employee of: Ethypharm Digital Therapy, F. Gheysen Consultant of: Ethypharm Digital Therapy, E. Haffen Consultant of: Ethypharm Digital Therapy, D. Drapier Consultant of: Ethypharm Digital Therapy, L. Lecardeur Consultant of: Ethypharm Digital Therapy

